# Novel Bis(4-aminophenoxy) Benzene-Based Aramid Copolymers with Enhanced Solution Processability

**DOI:** 10.3390/nano14201632

**Published:** 2024-10-11

**Authors:** Wonseong Song, Amol M. Jadhav, Yeonhae Ryu, Soojin Kim, Jaemin Im, Yujeong Jeong, Vanessa, Youngjin Kim, Yerin Sung, Yuri Kim, Hyun Ho Choi

**Affiliations:** 1 Department of Materials Engineering and Convergence Technology, Gyeongsang National University, Jinju 52828, Republic of Korea; 2 Research Institute of Green Energy Convergence Technology, Gyeongsang National University, Jinju 52828, Republic of Korea

**Keywords:** aramid copolymer, thin film, extreme environment, bar coating, polymerization

## Abstract

Aramid copolymers have garnered significant interest due to their potential applications in extreme environments such as the aerospace, defense, and automotive industries. Recent developments in aramid copolymers have moved beyond their traditional use in high-strength, high-temperature resistant fibers. There is now a demand for new polymers that can easily be processed into thin films for applications such as electrical insulation films and membranes, utilizing the inherent properties of aramid copolymers. In this work, we demonstrate two novel aramid copolymers that are capable of polymerizing in polar organic solvents with a high degree of polymerization, achieved by incorporating flexible bis(4-aminophenoxy) benzene moieties into the chain backbone. The synthesized MBAB-aramid and PBAB-aramid have enabled the fabrication of exceptionally thin, clear films, with an average molecular weight exceeding 150 kDa and a thickness ranging from 3 to 10 μm. The dynamic mechanical analysis (DMA) and thermogravimetric analysis (TGA) reveal that the thin films of MBAB-aramid and PBAB-aramid exhibited glass transition temperatures of 270.1 °C and 292.7 °C, respectively, and thermal decomposition temperatures of 449.6 °C and 465.5 °C, respectively. The mechanical tensile analysis of the 5 μm thick films confirmed that the tensile strengths, with elongation at break, are 107.1 MPa (50.7%) for MBAB-aramid and 113.5 MPa (58.4%) for PBAB-aramid, respectively. The thermal and mechanical properties consistently differ between the two polymers, which is attributed to variations in the linearity of the polymer structures and the resulting differences in the density of intermolecular hydrogen bonding and pi-pi interactions. The resulting high-strength, ultra-thin aramid materials offer numerous potential applications in thin films, membranes, and functional coatings across various industries.

## 1. Introduction

The aerospace and defense sectors, among others, increasingly use aramid, a polymer material that is known for its strong mechanical and thermal resistance, due to its reliable qualities in harsh settings [1,2,3,4]. Aramid is an amide-based synthetic polymer in which more than 85% of the amide groups (-CONH-) are directly linked to two aromatic groups [5,6]. The term ‘aromatic polyamide’ is used to distinguish it from aliphatic polyamides such as nylon. In general, aramids are used in fiber form due to their remarkable thermal properties, which include stability at temperatures above 400 °C and continuous use at 200 °C. As a result, aramids are high-strength, heat-resistant materials that are ideal for applications such as bulletproof vests and firefighter suits.

DuPont’s development of NOMEX in 1968, the first commercial meta-aramid fiber, received praise for its superior chemical resistance despite criticism for its poorer tensile quality [7,8]. In response, DuPont introduced Kevlar, a para-aramid fiber, in 1972. With its great strength, high elasticity, and chemical resistance, Kevlar has found widespread applications in sports equipment, tires, bulletproof vests, and other high-value textiles. [9,10]. In addition to Kevlar, Twaron (Teijin) is another well-known para-aramid. However, the rigidity of their polymer molecular structures and the strong hydrogen bonding between amide groups prevent these polymers from polymerizing in polar organic solvents like N-Methyl-2-pyrrolidone (NMP) or dimethylacetamide (DMAc). Instead, only strong acidic conditions like sulfuric acid can polymerize these polymers.

The new aramid copolymer structure that Teijin made avoided these problems by adding flexible links, like ether groups (-O-), to the traditional aramid framework. This approach was chosen so that fiber could be made in polar organic solvents for polymerization (Technora). This aramid copolymer is more stable than the usual methods that use sulfuric acid and allows for the use of solution spinning methods that use organic solvents [11,12]. As a specialty fiber, Technora is often more expensive than traditional aramids or synthetic fibers, which can limit its use in cost-sensitive applications. While it offers excellent thermal stability, it may not perform as well as conventional aramids like Kevlar in extreme high-temperature environments. Its high tensile strength is tempered by a degree of flexibility, which may be a disadvantage in applications requiring rigidity. Despite its decent chemical resistance, Technora may not withstand certain harsh chemicals as effectively as other aramids, which could restrict its use in specific environments. Additionally, its relatively limited availability compared to more common aramids can create supply chain challenges. Technora’s unique properties often require specialized processing techniques, increasing both the manufacturing complexity and costs. Similar to other synthetic fibers, the production and disposal of commercialized aramids raise environmental concerns regarding sustainability and waste management. Finally, its limited compatibility with certain resins or materials may complicate its use in composite applications. These limitations highlight the need for careful evaluation when selecting commercialized aramids for specific applications, as well as the importance of ongoing research to enhance its properties and expand its potential uses.

The remarkable characteristics of these materials are attributed to the crystallization of rigid aromatic amide bonds and the exceptionally high cohesive energy density created through the formation of highly directed and efficient interchain hydrogen bonding [13,14]. In addition, they are to blame for the fully aromatic polyamides’ insolubility [15], a disadvantage that limits the range of applications for these materials and raises research interests in the area of improved solubility. that forms the basis of the aramid structure. Materials possessing a high propensity for In recent years, a great deal of work has been carried out to design the rigid aromatic backbone’s chemical structure in order to produce aromatic polyamides that can be handled conventionally [16,17,18,19]. Ether linkages are known to increase the processability of aromatic polymers without significantly decreasing their thermal stability [20,21,22,23,24,25,26,27].

In this work, we aim to introduce novel aramid copolymers that are capable of being synthesized at high molecular weights in organic solvents. To achieve this, the flexibility of the polymer chain’s backbone was enhanced by introducing substituent bis(4-aminophenoxy) benzene monomers (BABs), leading to the high solubility of this organic solvent, even for a high-molecular-weight aramid copolymer. The synthesized doped solution enabled the manufacturing process of extremely thin, transparent films. Despite their low thickness, the measured thermal and mechanical properties were similar to or superior to those of previously reported aramid films.

## 2. Experimental Procedure

*Materials*: All chemicals utilized in the experiments were of analytical grade. N-methyl-2-pyrrolidone (NMP, 99.99% purity, Changxin, China), calcium hydroxide (Ca(OH)_2_, 97.10% purity, Acros, Geel, Belgium), terephthaloyl chloride (TPC, 99.90% purity, Shandong Kaisheng new Materials Co. Ltd., Zibo, China), p-phenylene diamine (PPD, 98.99% purity, TCI, Tokyo, Japan), 1,3-bis(4-aminophenoxy) benzene (MBAB, 97.40% purity, TCI), and 1,4-bis(4-aminophenoxy) benzene (PBAB, 99.50% purity, TCI) were purchased commercially and subsequently dehydrated before use. The anhydrous NMP with a water content below 80 ppm was created using an organic solvent dehydrating and deoxidation system (C&T International Co., Ltd., Incheon, Republic of Korea). All monomers were dehydrated by vacuum-drying overnight (SH SCIENTIFIC, Sejong-si, Republic of Korea).

*Polymerization*: The polymerization was conducted using a PBAB or MBAB: PPD: TPC molar ratio of 1:1:2 based on the polycondensation reaction. For example, the jacketed reaction flask was initially charged with 8.77 g of MBAB (or PBAB) and 3.24 g of PPD. After adding 300 g of NMP to the flask, the components were mixed at 250 rpm for 30 min (Step I: Dissolution). The initiation of polycondensation was marked by adding 12.18 g of TPC to the jacket, and the reaction was maintained at 25 °C for 0.5 to 2 h until the torque applied to the stirrer reached saturation (Step II-1: Polymerization at a low temperature). The temperature was then increased to 80 °C with a heating rate of ~1 °C/min with stirring (Step II-2: Polymerization during elevation of temperature). Finally, the solution was neutralized through the addition of 4.44 g of Ca(OH)_2_ until the pH of the doped solution reached ~7.

*Characterization*: Nuclear magnetic resonance (NMR, Bruker-300 spectrometer at 300 MHz, Germany) spectra were obtained using a Bruker-300 spectrometer at 300 MHz for ^1^H in a deuterated sulfuric acid solution (H_2_SO_4–_d_2_). Fourier-transform infrared (FT-IR, Bruker Vector 22 spectrometer, New York, NY, USA) spectra were recorded on a Bruker Vector 22 spectrometer with a resolution of 4 cm^−1^ across a range of 400–4000 cm^−1^. Dynamic mechanical analysis (DMA, DMA Q50, TA Instruments, New Castle, DE, USA) was performed using a TA Instruments DMA Q800, with a heating rate of 5 °C/min and a load frequency of 1 Hz in film tension geometry, where *T*_g_ was identified at the peak temperature of tan δ. Thermogravimetric analysis (TGA, TGA Q50, TA Instruments, USA) was conducted using a TA Instruments 2050, with a heating rate of 10 °C/min under a nitrogen atmosphere. The tensile strength and elongation at break were measured using a Tensile Tester for Paper and Board (HK 202E, Hengke Inc., Changzhou, China) at a speed of 25 mm/min at room temperature.

## 3. Results and Discussion

**Scheme 1:**
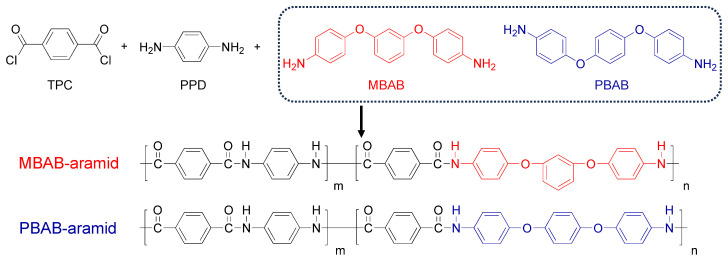
Molecular structures of aramid copolymers (MBAB-aramid and PBAB-aramid) and monomers used in polymerization. PPD: p-phenylenediamine, TPC: terephthaloyl chloride, MBAB: 1,3-bis(4-aminophenoxy) benzene, and PBAB: 1,4-bis(4-aminophenoxy) benzene.

**Figure 1:**
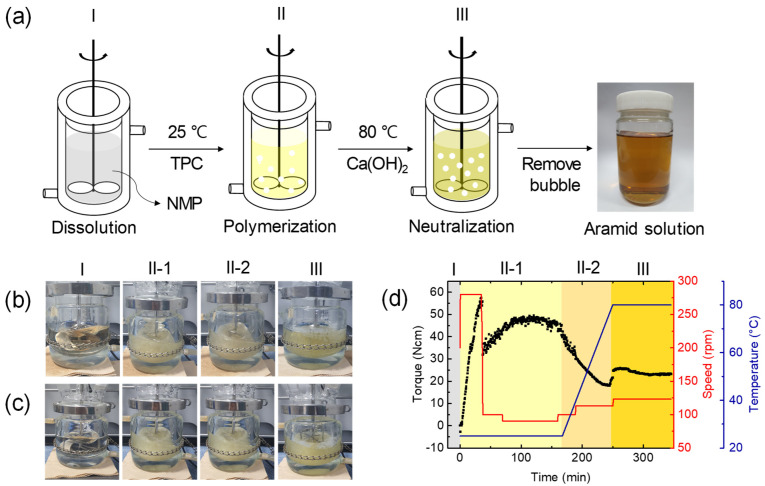
Organic solvent-based polymerization process of aramid copolymers. I: Dissolution of diamine, II: Polymerization (II-1: low temperature, II-2: elevation of temperature), and III: Neutralization. (**a**) Process scheme and photograph of doped solution. (**b**,**c**) Solution images as function of process step (panel b: MBAB-aramid, panel c: PBAB-aramid). (**d**) Torque (black dots) and rotation speed (red line) applied to stirrer and reactor temperatures (blue line) during entire polymerization steps.

The aramid molecular structure and synthesis process in this study are depicted in Scheme 1 and Figure 1, respectively. To enable a polymerization process in organic solvents, we propose a copolymer structure in which one-third of the benzene rings in the main chain are linked with ether (-O-) linkages (Scheme 1). For this, we used derivatives of bis(4-aminophenoxy) benzene monomers (BABs), particularly MBAB and PBAB, which differ in the position of ether (-O-) linkages on the benzene ring. The introduction of relatively flexible BABs enabled a high degree of polymerization, even when conducting typical polycondensation reactions based on organic solvents. The scheme of polymerization is shown in Figure 1a. The process is based on a polycondensation reaction and consist of three steps: I—Dissolution of diamine, II—Polymerization, and III—Neutralization. In particular, the temperature was varied in Step II to achieve substantial fluidity of the solution during Step III. We used the following substeps: II-1—Polymerization at a low temperature and II-2—Polymerization at elevated temperature. As observed in Figure 1b,c, where the polymer solution climbs up the stirring rod over time in Step II-1, the viscosity of the solution increased rapidly with the duration of the stirring polymerization. Figure 1d displays the temperature of the reaction vessel and the torque measured on the stirrer throughout the polymerization process. The increase in torque as the polymerization advanced was indicative of the rising viscosity. A decrease in viscosity was observed when the temperature was raised for the neutralization process. Ultimately, the final doped solutions for dissolving the aramid copolymers, MBAB-aramid and PBAB-aramid, at 6 wt% were clear and free from impurities, which verified the solubility of the synthesized aramid copolymers in NMP. Note that the feeding ratio may not exactly correspond to the composition ratio within the actual polymer chain. However, we optimized the polymerization conditions to maximize the degree of polymerization. Although the molecular structures of all diamine monomers, i.e., MBAB, PBAB, and PPD, differ, all molecules contain the aminophenyl moiety, which is expected to exhibit similar reactivity with TPC. Therefore, it is considered that the composition ratio within the polymer chain is likely to be similar to the feeding ratio.

The molecular weights of these polymers were measured by gel permeation chromatography (GPC) after calibration with the standard polystyrene using NMP as the eluent, The number-average molecular weights (Mns) of the synthesized aramid copolymers are in the range of 149–190 kDa (Table 1). Achieving high strength and substantial heat resistance in aramid copolymers typically requires a significant degree of polymerization, which is achievable in this process. We found that both the monomer purity and moisture content critically influence the polymerization environment [28]. For instance, although the neutralizing agent Ca(OH)_2_ can be added at the beginning of polymerization, this leads to continuous moisture production during the process. This moisture forms hydrogen bonds with the -NH_2_ moiety of the diamine monomer, which acts as an impediment to the condensation polymerization reaction with TPC [29]. Therefore, the neutralizing agent was applied at the end of the polymerization step. Furthermore, the moisture content in the NMP solvent was controlled to be below 80 ppm during the polymerization reaction.

The viscosity of the synthesized doped solutions was measured as a function of the temperature. Both MBAB-aramid and PBAB-aramid showed the typical behavior of high-viscosity polymer solutions, with the viscosity decreasing with the increasing temperature. Additionally, clear Newtonian behavior was observed at low frequencies during dynamic viscosity measurements. Interestingly, despite its relatively lower molecular weight, PBAB-aramid consistently exhibited higher viscosity across all temperature ranges. This suggests that PBAB-aramid may possess linear, rigid polymer chains, likely due to differences in the monomeric skeletal structures of PBAB and MBAB.

**Figure 2:**
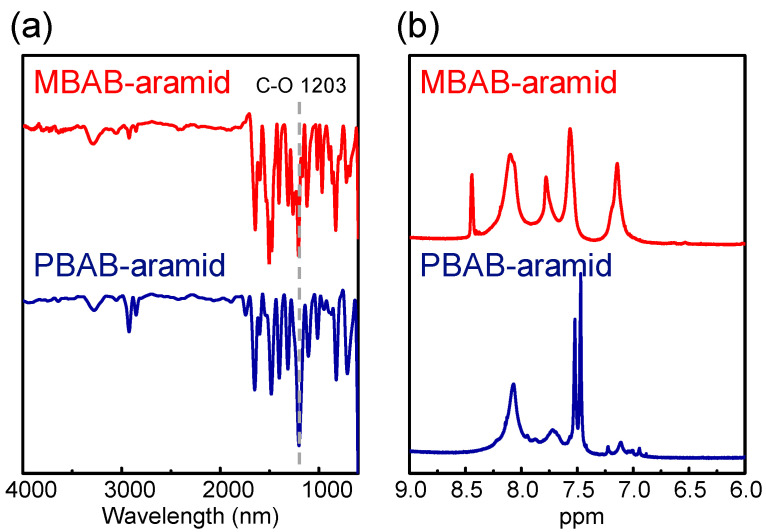
(**a**) FTIR and (**b**) ^1^H-NMR of MBAB-aramid (red line) and PBAB-aramid (navy line).

The structural features of the synthesized aramid copolymers were analyzed using FTIR spectra and ^1^H-NMR spectra (Figure 2a,b). The FTIR spectra of the aramid copolymers shown in Figure 2a displayed characteristic absorptions of the amide groups around 3200–3400 cm^−1^ (N–H stretching). The absorption band at 1600–1700 cm^−1^ is attributable to C=O stretching. Deformation coupling vibrations of C=N and N–H are responsible for the absorption bands at 1492 cm^−1^ and 1205 cm^−1^, respectively. Additionally, the absorption band at 1500–1538 cm^−1^ is attributed to C=C stretching vibrations of the aromatic ring. There is also an absorption band at 3000–2800 cm^−1^, caused by the C–H stretching vibration. In particular, the characteristic peaks from the ether linkages are clearly shown at 1203 cm^−1^ in both spectra, which have not been observed in Kevlar without ether linkages [30]. This confirms the inclusion of MBAB and PBAB monomers in the synthesized aramid copolymer. Figure 2b shows the ^1^H-NMR spectra of the aramid copolymers, showcasing proton signals attributed to benzene rings within the range of 7.00 to 8.20 ppm across all samples. A challenge in analyzing the spectra arises from the interaction between the solvent (D_2_SO_4_) and the terminal amine, as well as the amide groups within the polymer chains. This interaction leads to constant proton exchange, causing broadening and shifting of the solvent peak, which adds complexity due to peak overlap. However, a similar spectrum to that of traditional Kevlar-based aramid fibers was observed in the ^1^H-NMR, suggesting that the polycondensation resulting in the aramid structure was achieved [10].

**Figure 3:**
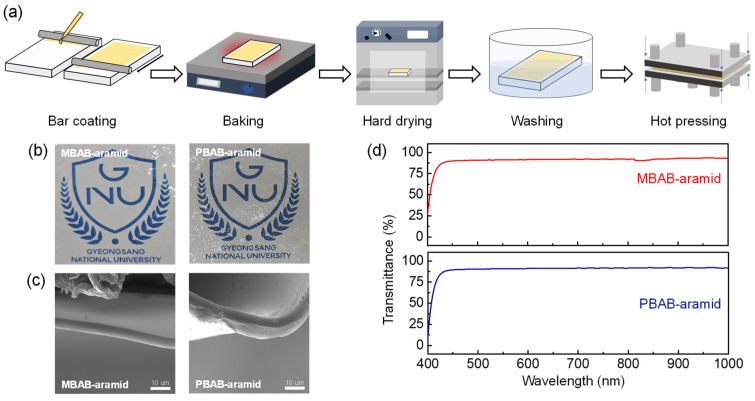
(**a**) Process of thin film fabrication. (**b**) Photographs and (**c**) SEM cross-section images of MBAB-aramid (left panel) and PBAB-aramid (right panel). (**d**) UV-vis-IR transmittance spectrum of 5 μm thick aramid films.

To fabricate free-standing films with a thickness of less than 10 μm over a large area, a bar coating process was employed using the synthesized doped solution (Figure 3a). A 10 cm × 10 cm glass substrate was placed onto the bar coater. Subsequently, 1 mL of the aramid doped solution was precisely applied to the corner of the glass substrate using a metal mill. During this phase, the coating speed was maintained at 20 mm/s. Following the application, the coated substrate was annealed on a hot plate at 120 °C. It is important to note that typical drying methods that rely on natural air-drying were not effective for creating uniform and clear aramid films. This sequence of application, coating, and annealing was repeated a total of five times. After the final annealing step, the substrate was placed in a vacuum at a maximum temperature of 80 °C for one day. Post vacuum treatment, the substrate was thoroughly rinsed by submerging it in distilled water for at least 12 h. This process removes CaCl_2_ salt, byproducts of the polymerization process, and NMP residue. Furthermore, films created without this additional process exhibit inferior properties in electrical insulation and surface stability, such as ion-induced electrical breakdown at extremely low voltages and time-dependent surface aging due to the aggregation of byproducts on the surface. To finalize the production process, the film was annealed under a hot press at 220 °C for 30 min.

Figure 3b presents a front and cross-sectional photograph of a free-standing aramid film with a thickness of 5–6 μm. As can be observed from the scanning electron microscope (SEM) image of the cross-section, the film exhibits a uniform density without any micrometer-sized phase separation or porosity. This indicates that the salts removed during the washing process were uniformly distributed within the film during the drying stage without aggregating. The front photograph demonstrates that the processed aramid film is highly transparent. Figure 3c shows the UV-vis-IR transmittance results for the manufactured aramid films, confirming that both films exhibit high transmittance, over 90% in the visible light region. Traditional aramid films have been produced by decomposing aramid fibers in a strong alkaline environment; however, due to the limitations of the alkaline decomposition method, the aramid molecules in the doped solution formed a nanofibrillar structure [31]. This nanofibrillar structure scatters light, resulting in an opaque film. However, in our doped solution, the aramid polymers are not dispersed as nanofibrils but are dissolved at the molecular level. Furthermore, during the drying process, they do not form nanostructures that could scatter light. Instead, they form a film with an amorphous-like structure, which we believe contributes to its high transparency.

The surface energy of the two polymer films was measured through contact angle measurements using water and diiodomethane as test liquids, with the contact angles, as well as the surface energy, being summarized in Table 2. The surface energies of MBAB-aramid and PBAB-aramid were calculated using the Owens, Wendt, Rabel, and Kaelble model (OWRK model) to be 48.2 mJ/m² and 48.4 mJ/m², respectively [32,33]. This value is similar to the previously reported surface energy of meta-aramid [34]. Due to the hydrophilic nature of the hydrogen bonds that are present in the aramid polymer chains, the water contact angles were found to be lower compared to hydrophobic polymers such as polystyrene and polyethylene [35]. In addition, the similar molecular structures and identical chemical compositions of the two polymers are believed to contribute to their similar surface energies.

**Figure 4:**
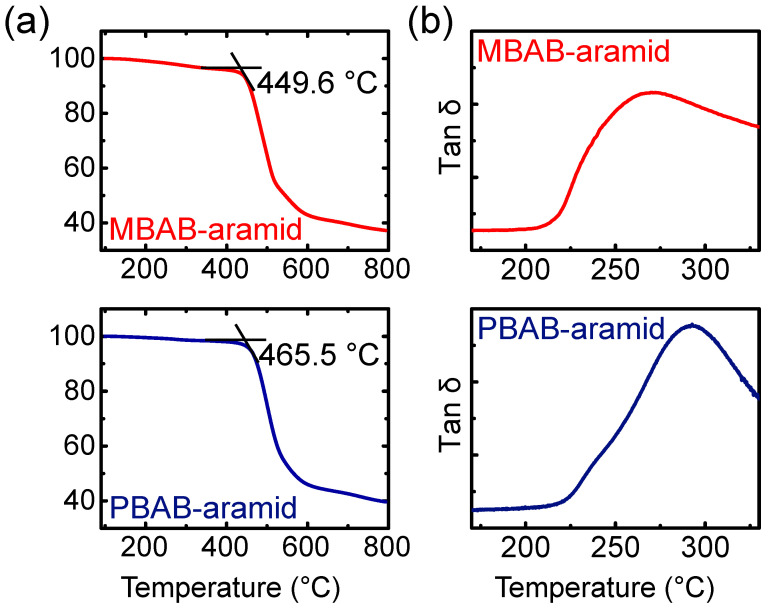
(**a**) Thermogravimetric analysis (TGA) and (**b**) dynamic mechanical analysis (DMA) of MBAB-aramid and PBAB-aramid films.

A thermogravimetric analysis (TGA) and a dynamic mechanical analysis (DMA) were conducted to determine the thermal stability and glass transition temperatures of films made from MBAB-aramid and PBAB-aramid (Figure 4). As indicated by the TGA results, shown in Figure 4a, both films exhibited stable behavior below 440 °C. The absence of mass loss at 100 °C indicates that despite the numerous hydrogen bonding units in the synthesized aramid polymer, there is no water present within the film. The absence of mass loss at 250 °C, considering that PPD and TPC can vaporize at this temperature, signifies that there are no monomer residues within the film. The onset points of thermal decomposition (*T*_onset_) for MBAB-aramid and PBAB-aramid were observed at 449.6 °C and 465.5 °C, respectively. The DMA measurements showed that the glass transition temperatures (*T*_g_) for MBAB-aramid and PBAB-aramid were 270.1 °C and 292.7 °C, respectively (Figure 4b). The higher *T*_onset_ and *T*_g_ of PBAB-aramid, relative to those of MBAB-aramid, are attributed to the linear rigidity of the PBAB-aramid polymer chains, as analyzed in previous rheological results. Aramid polymers are known to enhance thermal stability and mechanical strength through intermolecular hydrogen bonding between polymer chains [36]. When polymer chains possess linear rigidity, they can form stronger hydrogen bonds with the surrounding molecules. The higher linearity in the molecular structure of PBAB-aramid, compared to MBAB-aramid, results in a denser network of intermolecular hydrogen bonds, thereby contributing to its superior thermal stability.

**Figure 5:**
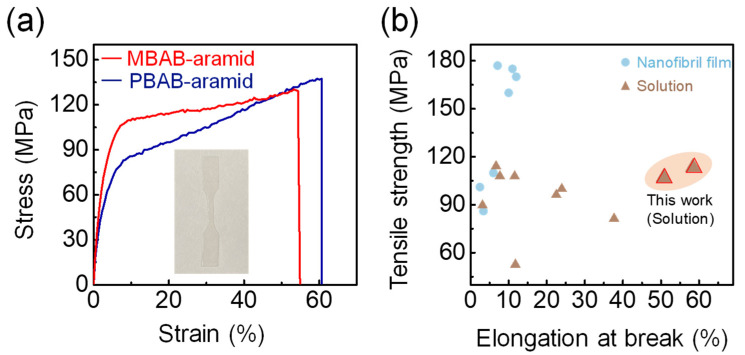
(**a**) Tensile properties of aramid films (inset: sample photograph). (**b**) Comparison of mechanical properties of aramid films in this work with previous aramid films [11,36,37,38,39,40,41,42,43,44,45].

The tensile properties of the film were measured using a total of five samples, and the average values are presented in Table 2. Figure 5 illustrates the stress–strain curve for the specimen that demonstrated the highest tensile strength. As indicated in the graph in Figure 5, the tensile strengths for MBAB-aramid and PBAB-aramid were 130.8 MPa (average 107.1 MPa) and 138.2 MPa (average 113.5 MPa), respectively. The elongation at break was also significant, with MBAB-aramid showing 54.9% (average 50.7%) and PBAB-aramid 61.3% (average 58.4%).

It should be noted that the differences in thermal and mechanical properties between MBAB-aramid and PBAB-aramid thin films were not as significant as we had anticipated. However, as shown in Table 2, the thermal properties (*T*_d5_, *T*_onset_, *T*_g_) and mechanical properties (tensile strength, elongation at break) consistently measured higher for the PBAB-aramid thin film, leading us to believe that the properties of the two films are not similar. This is likely due to the molecular structure of PBAB-aramid, which more readily forms a one-dimensional linear arrangement, whereas MBAB-aramid tends to form a coiled structure. The linear molecular structure facilitates denser intermolecular hydrogen bonding and pi-pi interactions, which explains the relatively higher thermal and mechanical properties of PBAB-aramid.

Figure 5b is a graph comparing the mechanical properties of previously developed aramid films [11,37,38,39,40,41,42,43,44,45,46]. Aramid films are categorized into nanofibril films, which are produced by breaking down Kevlar aramid fibers into nanofibril aggregates, and solution films, which are fabricated by casting a fully dissolved aramid doped solution, similar to our study. As shown in Figure 5b, the BAB-aramid film developed in our study exhibited tensile strength values that are similar to the highest values shown by prior solution films. Interestingly, the elongation at break demonstrated significantly superior performance compared to aramid films developed previously. This is believed to be due to the increased flexibility from the ether linkages contained in BABs. The relatively lower elongation at break in MBAB-aramid is thought to be due to the high-density entanglement between polymer chains caused by the molecule’s coiled structure.

## 4. Conclusions

In this work, new aramid copolymers enabling a high degree of polymerization under organic solvent process have been demonstrated. In the polymer chain’s backbone, one-third of the benzene rings in the main chain are chemically connected with flexible ether linkages by introducing BAB derivatives, MBAB, and PBAB as comonomers. By conducting the polycondensation process in a moisture-controlled and purity-regulated environment, we were able to maximize the degree of polymerization through real-time observation of the torque. The synthesized copolymers, MBAB-aramid and PBAB-aramid, with an average molecular weight exceeding 150 kDa, have enabled the fabrication of exceptionally thin, clear films, with controllable thicknesses ranging from 3 to 10 μm and average transmittance exceeding 90%. The decomposition temperature and the tensile strengths, with elongation at break, are slightly higher in PBAB-aramid (465.5 °C and 113.5 MPa (58.4%)) than in MBAB-aramid (449.6 °C and 107.1 MPa (50.7%)), which can presumably be attributed to variations in the linearity of the polymer structures and the resulting differences in the density of intermolecular hydrogen bonding and pi-pi interactions. Interestingly, both aramid films demonstrated tensile strengths that are comparable to the highest values reported for previous solution films. Moreover, they showed a significantly improved elongation at break, indicating the enhanced flexibility provided by the ether linkages in BABs. These distinct properties and the ease of film processing compared to traditional aramid fibers and films suggest potential applications in various fields such as insulating thin films, membranes, and functional coatings for aramid materials.

## Figures and Tables

**Table 1:** Molecular weights and viscosity properties of the synthesized aramid copolymers.

Sample	Mn (g/mol)	Mw (g/mol)	Viscosity (Pa·s)		
			30 °C	50 °C	80 °C
MBAB-aramid	190,000	495,000	193.9	91.0	35.2
PBAB-aramid	149,000	432,000	194.3	98.7	42.7

**Table 2:** Surface energy, thermal stability, and average mechanical properties with standard deviation of aramid films with thickness of 5 μm. The average and standard deviation are results analyzed from measurements of five samples. ( ): maximum value.

Properties		MBAB-Aramid	PBAB-Aramid
Surface energy (mJ/m^2^)	Polar	39.0	34.1
	Dispersive	9.3	14.3
	Total	48.2	48.4
*T*_d5_ (°C)		451.3	459.3
*T*_onset_ (°C)		449.6	495.5
*T*_g_ (°C)		270.1	292.7
Tensile strength (MPa)		107.1 ± 21.2 (130.8)	113.5 ± 23.1 (138.2)
Elongation at break (%)		50.7 ± 4.2 (54.9)	58.4 ± 14.8 (61.3)
Modulus (GPa)		3.99 ± 0.60 (4.39)	2.95 ± 0.98 (3.07)

## Data Availability

The original contributions presented in the study are included in the article, further inquiries can be directed to the corresponding author/s.
